# Patient With Advanced Aggressive B2 Thymoma Achieved Positive Outcomes Post CAP‐Endostar Combination Therapy

**DOI:** 10.1111/crj.70081

**Published:** 2025-06-22

**Authors:** Min Zhang, Jingjing Zhang, Kelei Zhao, Xiaohan Yuan, Jinghang Zhang, Yanting Liu, Ping Lu

**Affiliations:** ^1^ Department of Oncology The First Affiliated Hospital of Xinxiang Medical University Weihui China; ^2^ Department of Pathology The First Affiliated Hospital of Xinxiang Medical University Weihui China

**Keywords:** Endostar (recombinant human endostatin injection), radiation therapy, thymic tumors

## Abstract

This case report features a patient with an invasive thymoma. The patient presented with an anterior mediastinal mass that invaded the left brachiocephalic trunk vein, resulting in the formation of a carcinoma thrombus in the right atrium, superior vena cava, left brachiocephalic trunk vein, and left internal jugular vein (Masaoka stage IV). No indication for surgery was assessed by surgical consultation. After six cycles of chemotherapy and chest radiotherapy, the tumor size decreased from 72.43 to 24 mm, showing a significant improvement in patient efficacy. After a follow‐up of 50 months, the patient remained well, without local recurrence or distal metastasis, and maintained a partial response (PR).

## Graphical abstract

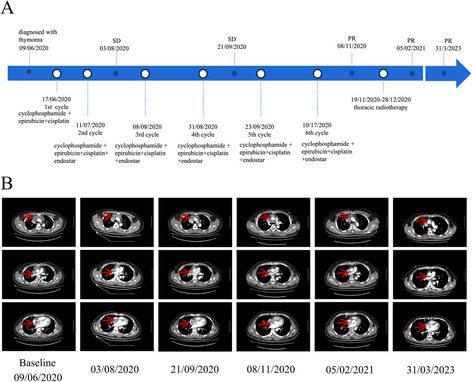

## 1 Introduction

Thymoma is a rare mediastinal tumor that originates from the epithelial cells of the thymus. The annual incidence of thymoma in China is 0.17 per 100 000 individuals [1]. According to the criteria published by the World Health Organization (WHO), thymomas can be classified into different histologic subtypes, such as A, AB, B1, B2, B3, and other rare types. The low‐risk and high‐risk groups include types A/AB/B1 and B2/B3, respectively [2]. Currently, surgical intervention remains the primary treatment approach, while systemic chemotherapy is recommended for unresectable, recurrent, or metastatic thymomas. Here, we report an exceptionally rare case of advanced B2 aggressive malignant thymoma that invaded the left brachiocephalal trunk vein and right atrial, superior vena cava, left brachiocephalal trunk vein, and left internal jugular vein formation (Masaoka stage IV) treated with CAP‐Endostar regimen. According to the 2020 Chinese Society of Clinical Oncology (CSCO) guidelines and some studies, patients with stage TV thymoma can be treated with the combination regimens including anti‐angiogenensis drugs, such as sunitinib, bevacizumab, or endostatin. Considering the strong therapeutic intent of the patient and the higher tumor burden, a combined regimen including anti‐angiogenensis drugs was chosen. However, due to the elevated bleeding risk associated with sunitinib and bevacizumab, endostatin emerges as the optimal therapeutic choice.

## 2 Case Description

**FIGURE 1:**
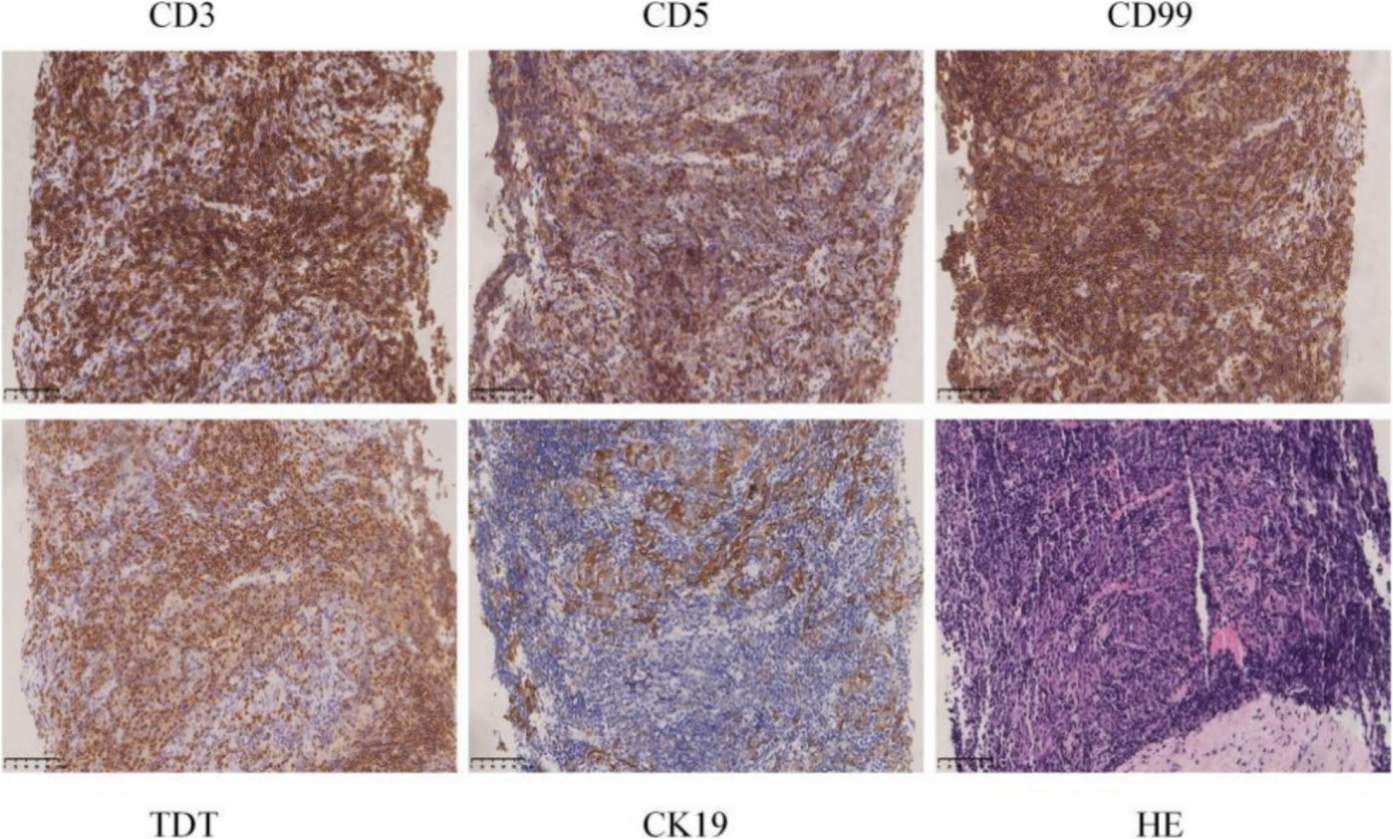
Needle biopsy specimens of the mediastinal mass and pathological images of HE‐stained specimens.

**FIGURE 2:**
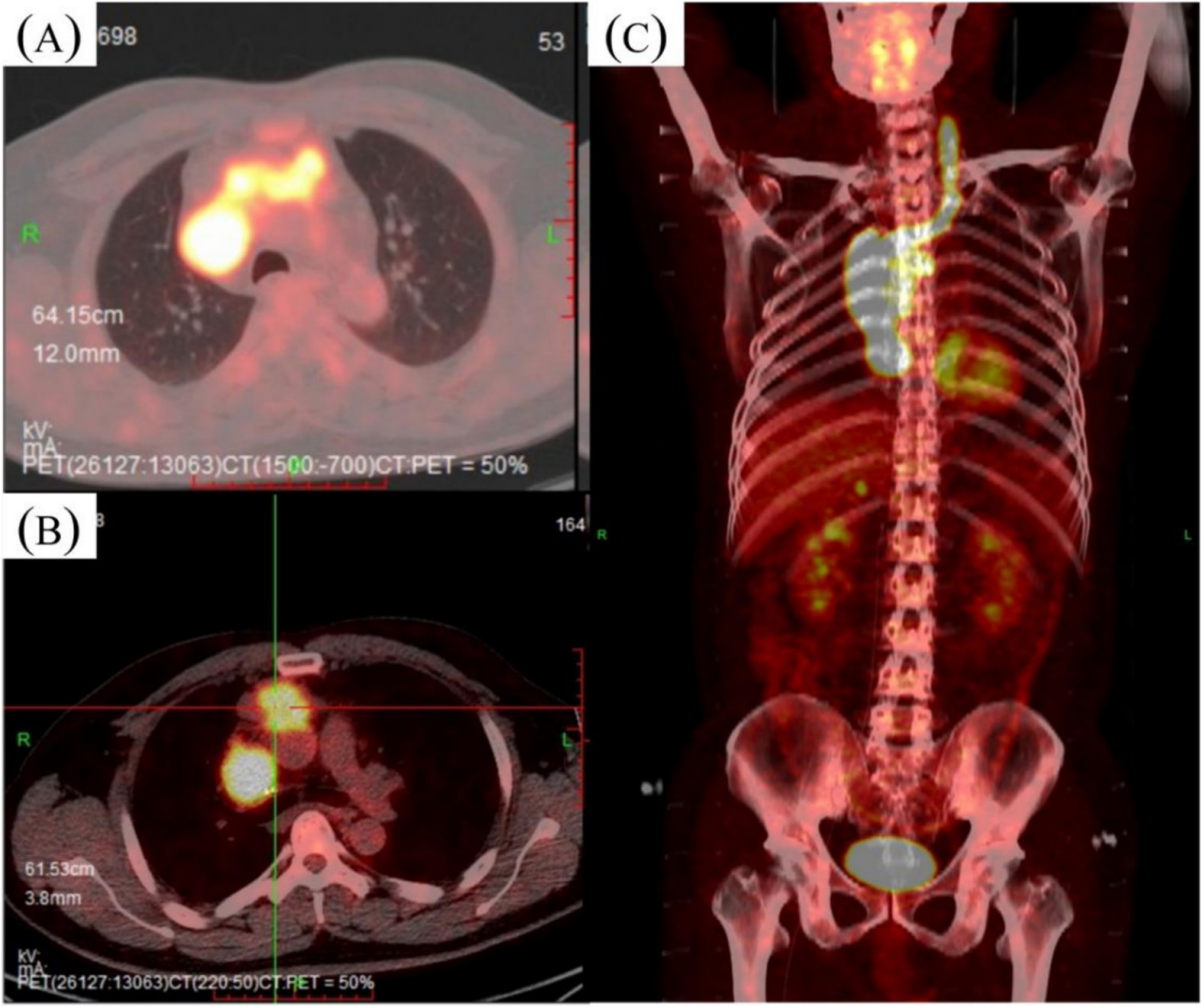
PET‐CT images of the case. (A,B) Images showing thoracic mediastinal window treatment. (C) Local PET‐CT images of the coronal plane.

A 40‐year‐old man presented with obvious double eyelid edema occurring in the morning and relieved spontaneously, with gross hematuria. In 2020, an ultrasound‐guided needle biopsy was performed, revealing pathological findings consistent with thymoma type B2 (Figure 1). The chest PET/CT scan showed an invasion of thymic malignancy to the left brachiocephalic vein, forming a tumor in the right atrium, superior vena cava, left brachial vein, and left internal jugular vein with mediastinal metastasis (Masaoka stage IV) (T3N1M1b, IVB) (Figure 2). A cardiac ultrasound echo was performed on June 17, 2020, which revealed a hypoechoic area in the right atrium, suggestive of an embolus. Consultation of thoracic and cardiac surgery did not suggest any surgical treatment or thrombectomy indication. Following evaluation by the radiotherapy physician, it was determined that the symptoms of superior vena cava syndrome of the patient carried a risk of exacerbating edema. Additionally, if the tumors were to regress quickly, there was a potential risk of massive bleeding.

**FIGURE 3:**
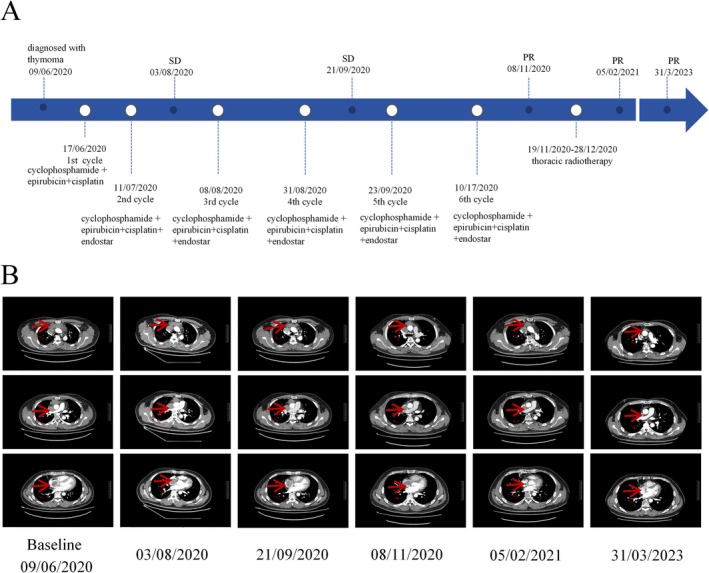
(A) Timeline of the case. (B) Image changes of chest CT in three different planes during treatment.

In accordance with the relevant guidelines, the patient received Period 1 of CAP (cyclophosphamide 500 mg/m^2^, epirubicin 50 mg/m^2^ IVd1, cisplatin 50 mg/m^2^ IVd1, q21day) chemotherapy regimen on June 17, 2020. During the interval of chemotherapy, the patient experienced myelosuppression of Grade II; however, the indications subsequently normalized following ascending leukocyte treatment. Due to tumor infiltration in major blood vessels, the use of antiangiogenic drugs can mitigate the bleeding risk, while validation of the pertinent relevant data can enhance therapeutic efficacy. Recombinant human vascular endostatin was introduced on July 11, 2020, followed by a subsequent regimen of five treatment cycles on August 8, 2020, August 31, 2020, September 23, 2020, and October 17, 2020. The specific plan is as follows: CAP (cyclophosphamide 500 mg/m^2^ IVd1, epirubicin 50 mg/m^2^ IVd1, cisplatin50mg/m^2^IVd1, q21day) combined with Endosta therapy. Evaluation of the response after 2 and 4 of chemotherapy cycles revealed stable disease (SD) with a reduction in the size of the local lesion. Following a total of six chemotherapy cycles, a chest CT scan achieved a partial response (PR). Considering the presence of visible atrial and venous tumors, and intermittent facial edema, consultation with radiologists recommended chest radiotherapy. Following the exclusion of radiotherapy contraindications, the patient underwent chest radiotherapy on November 19, 2020. The type of radiotherapy was 3D intensity modulation and radiotherapy. First, 20 radiotherapy sessions of 2 Gy were administered to the primary lesion, followed by 10 sessions of 2 Gy to the lymph nodes after relocalization. During radiotherapy, the patient developed radiation esophagitis, which resolved after symptomatic treatment. At the 50 months of follow‐up, the patient remained in a stable condition without evidence of local recurrence or distal metastases (Figure 3) (Table 1).

**TABLE 1:** The case was the entire course of treatment and reviewed CT tumor size evaluation.

CT time	Tumor size (mm)	Efficacy assessment	Chemotherapy time	Chemotherapy regimen	Radiotherapy time	Radiotherapy dose
09/06/2020	72.43	Baseline	17/06/2020	CAP	19/11/2020–28/12/2020	2 Gyx 30 times
03/08/2020	55.2	SD	11/07/2020	CAP + anti‐angiogenic drugs		
21/09/2020	54	SD	08/08/2020	CAP + anti‐angiogenic drugs		
08/11/2020	50.6	PR	31/08/2020	CAP + anti‐angiogenic drugs		
05/02/2021	44	PR	23/09/2020	CAP + anti‐angiogenic drugs		
26/04/2021	36	PR	17/10/2020	CAP + anti‐angiogenic drugs		
05/09/2021	28.58	PR				
21/02/2022	32	PR				
08/07/2022	25	PR				
12/10/2022	25	PR				
09/01/2023	24	PR				
31/03/2023	23	PR				

Abbreviations: CAP, C: cisplatin; A: cisplatin; P: cisplatin; PR, partial response; SD, stable disease.

## 3 Discussion

Malignant thymoma (type B2) is a rare tumor characterized by pathological features indicative of malignancy and highly aggressive biological behavior. It can be associated with extrathoracic metastases to the liver, kidney, and lymph nodes [3]. The optimal treatment for malignant thymoma depends on the stage and extent of the disease which usually includes a combination of surgical resection, chemotherapy, and radiotherapy. However, given the rarity of advanced malignant thymomas, there is limited clinical data from large randomized trials to establish definitive treatment guidelines. Platinum‐based chemotherapy regimens combined with vincristine, doxorubicin, and etoposide have demonstrated efficacy in case of inoperable aggressive thymoma tumors [4]. Commonly used treatment options include anthracycline‐based regimens such as “cisplatin + doxorubicin + vincristine + cyclophosphamide” (ADOC) or “cisplatin + doxorubicin + cyclophosphamide” (PAC) [5]. The expression of angiogensis markers vascular endothelial growth factor (VEGF), VEGF receptor‐1, and VEGF receptor‐2 in thymomas (type B2) showed moderately positive (2+), intensely positive (3+), and moderately positive (2+), respectively. This suggested that angiogenesis is associated with the occurrence and development of thymomas (type B2); thus VEGF, VEGFR1 and VEGFR1 maybe the prognostic and prognostic markers for anti‐angiogenesis therapies [6]. Several studies have shown that compared with CAP chemotherapy alone, chemotherapy combined with targeted therapy such as sunitinib, anti‐angiogenic drugs bevacizumab, or Endostar can improve objective response rate (ORR) [7]. This combination therapy may prolong OS in patients with thymic carcinoma or thymoma. However, the current study results did not achieve statistical significance (*p* = 0.348), likely due to the small sample size. The recombinant human endostatin, Endostar can inhibit angiogenesis and tumor growth. Considering the presence of factors such as vascular invasion, vascular wrapping, urgency and patient safety, the treatment strategy of CAP combined with Endostar was selected to achieve PR, and ensure good safety, thereby creating conditions for subsequent local treatment. Chemotherapy (CH) is usually used as either induction or definitive treatment in cases when the tumor is deemed unresectable [8]. Radiotherapy (RT) is applied to selected patients, especially as adjuvant therapy. A multimodal approach is beneficial for a group of patients with aggressive tumors. The 5‐year survival rate of thymoma patients with distant metastasis varies between 13.3% and 81% following multimodal treatment, including surgical resection, pleurectomy, chemotherapy, and radiotherapy [9].

Here, we report a middle‐aged male patient with advanced aggressive malignant thymoma. Remarkably, the patient achieved long‐term survival after a treatment with a systemic therapy regimen (CAP in combination with endostatin) and local radiotherapy. This case brings us the following inspirations: (1) The combination of CAP chemotherapy with endostatin demonstrated efficacy and safety in treating stage IV thymoma (type B2) with higher tumor burden. (2) Radiation therapy may be the optimal treatment to control the disease, prolong the survival and improve the quality of life, when the disease progresses locally.

This successful outcome underscores the efficacy of combining these two therapeutic modalities in managing advanced cases of aggressive malignant thymoma.

In addition, this case also has some deficiencies. Immunohistochemistry (IHC) failed to demonstrate expression of VEGF, VEGFR1 and VEGFR2. The biopsy tissues were unsuitable for additional biomarker analysis by immunohistochemistry assay, because the tissues had been stored for more than 3 years.

## Author Contributions

Min Zhang, Xiaohan Yuan, and Yanting Liu, and Ping Lu conceptualized and designed the study and revised the manuscript. Jingjing Zhang, Kelei Zhao, and Jinghang Zhang collected, organized, and analyzed clinical, genetic, and pathologic data and were responsible for manuscript writing. All authors contributed to the manuscript and approved the final version.

## Ethics Statement

This research was in accordance with the Declaration of Helsinki and approved by the ethics committee of the First Affiliated Hospital of Xinxiang Medical University [EC‐022‐114]. The patient had signed the informed consent voluntarily.

## Consent

Written informed consent from the patient for the use of figure and publication of their case details has been obtained by the authors.

## Conflicts of Interest

The authors declare no conflicts of interest.

## Supporting information


**Data S1** Supplementary Information.

## Data Availability

The data that support the findings of this study are available on request from the corresponding author. The data are not publicly available due to privacy or ethical restrictions.
