# Supplementary figures and images for: Patient With Advanced Aggressive B2 Thymoma Achieved Positive Outcomes Post CAP‐Endostar Combination Therapy
Source: Clin Respir J. 2025 Jun 22;19(6):e70081. doi: 10.1111/crj.70081 (PMC12182910; doi:10.1111/crj.70081)

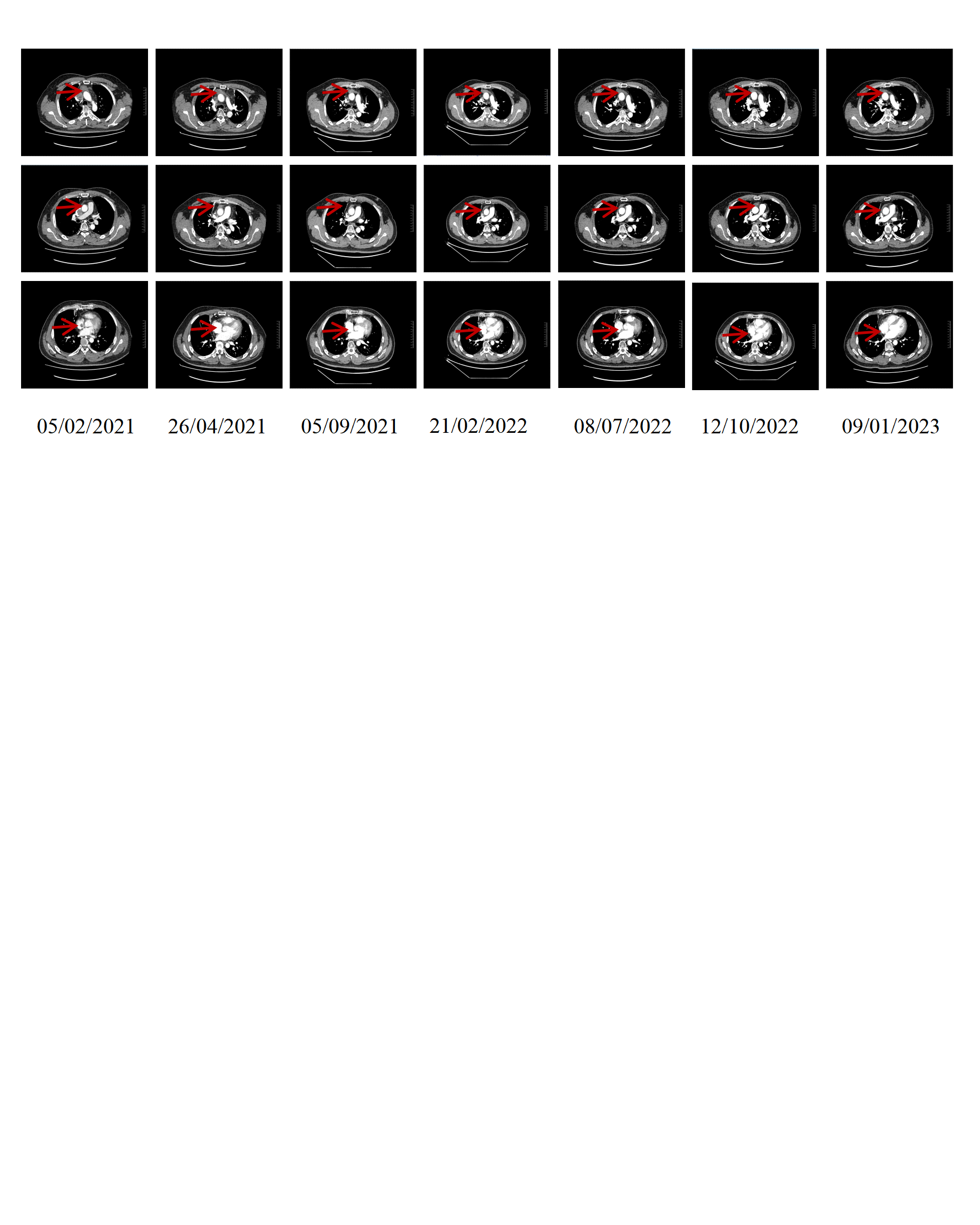

Supplement: Supplementary file 1 — Data S1 Supplementary Information. [file CRJ-19-e70081-s001.tif]
